# Cardiovascular disease risk factors in a Nigerian population with impaired fasting blood glucose level and diabetes mellitus

**DOI:** 10.1186/s12889-016-3910-3

**Published:** 2017-01-06

**Authors:** Victor M. Oguoma, Ezekiel U. Nwose, Ifeoma I. Ulasi, Adeseye A. Akintunde, Ekene E. Chukwukelu, Phillip T. Bwititi, Ross S. Richards, Timothy C. Skinner

**Affiliations:** 1 School of Psychological and Clinical Sciences, Charles Darwin University, Darwin, NT 0909 Australia; 2 School of Community Health, Charles Sturt University, Orange, NSW Australia; 3 Department of Public and Community Health, Novena University, Ogume, Delta State Nigeria; 4 College of Medicine, University of Nigeria and University of Nigeria Teaching Hospital, Nsukka, Nigeria; 5 Department of Internal Medicine, Ladoke Akintola University of Technology, Ogbomoso, Oyo State Nigeria; 6 Department of Chemical Pathology, College of Medicine, University of Nigeria Teaching Hospital, Ituku Ozalla, Nigeria; 7 School of Biomedical Sciences, Charles Sturt University, Wagga Wagga, NSW Australia

**Keywords:** Diabetes, Co-morbidity, CVD risk factors, Impaired fasting glucose, Nigeria, Prediabetes

## Abstract

**Background:**

Diabetes is a risk factor for cardiovascular diseases (CVDs) and there are reports of increasing prevalence of prediabetes in Nigeria. This study therefore characterised CVDs risk factors in subjects with impaired fasting glucose (IFG) and diabetes.

**Methods:**

Data from 4 population-based cross-sectional studies on 2447 apparently healthy individuals from 18 - 89 years were analysed. Anthropometric, blood pressure and biochemical parameters were collected and classified. Individuals with IFG (prediabetes) and diabetes were merged each for positive cases of dyslipidaemia, high blood pressure (HBP) or obesity. Optimal Discriminant and Hierarchical Optimal Classification Tree Analysis (HO-CTA) were employed.

**Results:**

Overall prevalence of IFG and diabetes were 5.8% (CI: 4.9 – 6.7%) and 3.1% (CI: 2.4 – 3.8%), respectively. IFG co-morbidity with dyslipidaemia (5.0%; CI: 4.1 – 5.8%) was the highest followed by overweight/obese (3.1%; CI: 2.5 – 3.8%) and HBP (1.8%; CI: 1.3 – 2.4%). The predicted age of IFG or diabetes and their co-morbidity with other CVD risk factors were between 40 – 45 years. Elevated blood level of total cholesterol was the most predictive co-morbid risk factor among IFG and diabetes subjects. Hypertriglyceridaemia was an important risk factor among IFG-normocholesterolaemic-overweight/obese individuals.

**Conclusion:**

The higher prevalence of co-morbidity of CVD risk factors with IFG than in diabetes plus the similar age of co-morbidity between IFG and diabetes highlights the need for risk assessment models for prediabetes and education of individuals at risk about factors that mitigate development of diabetes and CVDs.

**Electronic supplementary material:**

The online version of this article (doi:10.1186/s12889-016-3910-3) contains supplementary material, which is available to authorized users.

## Background

Impaired fasting glucose (IFG) is a hyperglycaemic condition that predisposes individuals to high risk of developing type II diabetes (T2D) and cardiovascular disease (CVD) [1]. It is characterised by insulin resistance, beta cell dysfunction, increased lipolysis, inflammation, sub-optimal incretin effect and hepatic glucose overproduction [2]. There have been debates on whether IFG, a component of prediabetes, should be considered in the same manner with T2D since in both conditions there is hyperglycaemic-induced toxicity [3, 4]. It is noted that progression from IFG to T2D can occur due to worsening oxidative stress [3], insulin resistance or beta cell dysfunction [4], and in the long term predisposes to increased risk of CVD. This is where routine screening can be beneficial in aiding early identification and application of management practices, since the process of progression is not abrupt.

In Africa, more than two thirds of people with diabetes are undiagnosed [5], giving room for complete transition of the early stages of impaired glucose regulation to overt disease condition. About 14.2 and 34.9 million adults of age 20 – 79 years have diabetes and prediabetes with estimated prevalence of 3.2% (2.1 – 6.7%) and 7.9% (4.8 – 21.9%), respectively. However, the most glaring part is the indication that the prevalence of prediabetes will double by the year 2040 and this is more than that projected for T2D [5]. Based on the 2013 Global Burden of Disease study [6], about 90.5% of stroke burden was attributable to lifestyle-related risk factors, cluster of metabolic risk factors (high blood pressure, IFG, elevated total cholesterol and low glomerular infiltration) and environmental factors. Lifestyle-related risk factors and metabolic risk factors contributed to 74.2% and 72.4% stoke burden, respectively and the population attributable fraction of risk factors in low-middle-income countries increased from 1990 to 2013 [6]. Such observations therefore call for periodic screening, monitoring and appraisal of data on risk factors for metabolic diseases.

CVD risk screening in Nigeria is sub-optimal, even though evidence indicates early onset of disease burden [7, 8], which in part contributes to the low life expectancy and quality of life of Nigerians [9]. One of the effective approaches to early identification of CVD risk is the utilisation of risk assessment models and these have been shown to be better than clinical judgement [10]. Although the assessment of co-occurrence of risk factors for CVD is largely studied in diabetic patients [11–14], the extent that these trends affect the seemingly healthy populations with IFG in Nigeria needs to be documented.

The use of risk assessment models, which provide the bedrock for early identification of diabetes and consequential CVD within a resource constraint population are unclear within African population [15]. Therefore, this study seeks to characterise CVD risk factors in subjects with impaired fasting glucose (IFG) and diabetes by specifically predicting the age of IFG and diabetes co-morbidity with CVD risk factors and to classify the risk factors in IFG in a large apparently healthy Nigerian population.

## Methods

### Study population, setting and ethics clearance

This was a consolidation of data from 4 population-based cross-sectional studies on apparently healthy individuals from 18 to 89 years in 4 geopolitical zones of Nigeria as reported [16]. Participants were drawn from a representative sample populations in Ndokwa West Local Government Area (LGA) of Delta State and indigenes of Ndokwa residing in Lagos State; Enugu North LGA and Enugu Municipality of Enugu State; and FCT Abuja. Participants gave informed consent and were recruited if they confirmed that they had no case or history of chronic kidney disease and diabetes and, were not pregnant or under treatment for high blood pressure.

In each of the study location, participants were drawn from both rural and urban settings. The main occupation of participants from rural populations is farming, while those in the urban setting are majorly employed as artisans, traders and in white-collar jobs. Yearly average temperature of study areas is 24^o^C (19 - 33^o^C). Participants were mobilised to attend designated study sites located at churches, town halls, school premises and primary health centres. Sample size was calculated based on two estimates since several CVD risk factors with different prevalence were involved. Firstly, estimated 5% prevalence of diabetes [17] at 5% confidence interval and at 95% confidence level gave a minimum sample size of 73. In regards to other CVD risk factors, minimum estimate of 384 sample size was also required given a projected 50% prevalence of hypertension and dyslipidaemia [18, 19] at 5% confidence interval and at 95% confidence level. However, the total sample size of 2447 in this study was above the minimum estimates for the various CVD risk factors assessed. The Human Research Ethics Committee of Charles Darwin University, Australia (HREC Ref: H14003); HREC of Novena University Nigeria. Ethics Review Committee (ERC) of the University of Nigeria Teaching Hospital, and ERC of Enugu State Ministry of Health. ERC of Benue State University Nigeria; Abuja Municipal and Kuje Area Councils Ethical Committees, approved the study.

### Anthropometric and blood pressure measurements

The standard techniques and procedures for measurement of waist circumference, weight and height were as reported [20–24]. The body mass index (BMI) was calculated by dividing the weight with height square (Kg/m^2^). BMI values were grouped into underweight (<18.5 Kg/m^2^), normal weight (18.5 – 24.9 Kg/m^2^), overweight (25.0 – 29.9 Kg/m^2^) and obese (>30 Kg/m^2^). Three readings of the systolic (SBP) and diastolic blood pressures (DBP) were taken using Omron® (Australia) digital and Accoson® mercury sphygmomanometer then the average of 2^nd^ and 3^rd^ readings was used to ascertain the blood pressure for all the study sites.

### Biochemical parameters

Participants fasted for at least 8 h before blood samples were collected for fasting blood glucose (FBG) and lipids analyses. Blood sample collections were carried out in different locations, such as town halls, health centres and churches, based on convenience of participants. EasyLog EL-USB-1® (Lascar Electronics, Australia) temperature data logger was used to monitor and ensure optimal operational temperature and humidity for point of care blood analysis. The temperature data logger was set 19.5^o^C low alarm and 30.5^o^C high alarm. For analysis using clinical laboratory techniques, blood sample were collected in fluoride oxalate bottles. Based on manufacturers’ guideline, the following screening techniques were used based on study location as earlier described [16].

*Fasting blood glucose*: CardioChek® Professional Point of Care Diagnostic Analyser (Polymer Technology Systems, Inc, IN, USA) [site 1] and Accuchek® Point of Care Diagnostic Analyser (Roche Diagnostics, UK) [site 2] were employed. At sites 3 and 4, FBG was assessed by the glucose oxidase method in the reference laboratories in Abuja and Enugu, Nigeria, respectively.

*Lipids profile*: Clinical laboratory techniques were used for screening total cholesterol (TC), triglycerides (TG) and high density lipoprotein cholesterol (HDL-C) at reference laboratories in sites 2, 3 and 4. TC and TG were measured by enzymatic colorimetric method and HDL-C by differential precipitation followed by enzymatic colorimetry using semi-autoanalyser Mitra Photometer (Linear Chemicals S.L., Barcelona, Spain [site 2], Randox Laboratories, UK [site 3] and Teco Diagnostics, USA [site 3]).

The point of care analytical systems (Polymer Technology Systems and Roche Diagnostics) are certified by the Centre for Disease Control and Prevention (CDC) to meet clinical laboratory reference standards [25]. In addition, CardioChek® Professional Point of Care Diagnostic Analyser has been shown to demonstrate good clinical agreement with clinical laboratory reference methods for TC (97%), TG (92.4%) and HDL-C (92.9%) [26].

### Classification of CVD risk factors

The blood glucose level range of 100 – 125 mg/dL (5.6 – 6.9 mmol/L) was used to define impaired fasting glucose (IFG) and values ≥126 mg/dL (7 mmol/L) for diabetes when participants fasted for at least 8 h. The cut-off values employed for the other CVD risk components are as proposed [27]:

- High blood pressure: Systolic blood pressure ≥140 mmHg and/or diastolic blood pressure ≥90 mmHg [28].
- Hypertriglyceridaemia: Serum triglyceride level of ≥150 mg/dL (1.7 mmol/L) and/or receiving current medication for the condition [29].
- Low HDL-C: Serum HDL-C level of ≤40 mg/dL (1 mmol/L) in males and ≤50 mg/dL (1.3 mmol/L) in females [29].
- Hypercholesterolaemia: Serum total cholesterol level of ≥200 mg/dL (5.2 mmol/L) and/or receiving current medication for the condition [29].

Dyslipidaemia was defined when at least one lipid disorder (hypertriglyceridaemia, hypercholesterolaemia and or low HDL-C) was identified.

### Statistical analysis

A total of 2809 individuals were pooled from the 4 population-based cross-sectional studies. Study participants missing values of key study variables such as FBG, TG, TC, HDL-C, BMI, and SBP/DBP were excluded. After the exclusion (*n* = 362), 2447 participants were used. Descriptive analysis of the study population was performed showing the mean and standard deviation of all CVD risk variables across gender. In order to determine number of participants with IFG/diabetes co-morbid CVD risk factors, participants with positive tests for IFG or diabetes were merged with positive cases of dyslipidaemia, HBP, overweight/obese (based on BMI).

Frequency distribution of IFG and diabetes with their co-morbid states were calculated and tabulated. Bootstrapping was applied based on 1000 bootstrap samples and 95% confidence interval generated.

Optimal Discriminant Analysis (ODA) was carried out to predict the age at which IFG or diabetes co-morbidity with CVD risk factors occur across the entire study population. In this analysis, IFG and diabetes with their co-morbid risk factors were treated as class variables, with age of participants as the attribute variable (See Additional file 1).

Finally, Hierarchical Optimal Classification Tree Analysis (HO-CTA) using Optimal Discriminant Analysis (ODA) methods [30] was applied to predict the risk factors that are associated with IFG as well as to generate a decision model. HO-CTA has been shown to be better than linear models that are based on legacy general linear model and maximum-likelihood paradigms [31]. It is a nonlinear multivariate classification method and has an advantage of not requiring any assumptions of multicolinearity, multivariate normality, equality of group sizes, or number of variables [32].

For the HO-CTA, IFG was the dependent (class) variable, while hypercholesterolaemia, hypertriglyceridaemia, HBP, low serum HDL-C level, age, overweight/obese (based on BMI), smoking and alcohol status, and population (rural vs. urban) were fitted as independent covariates or attribute variables. Leave-one-out re-sampling was used to identify the most stable predictor (attribute) variables that maximise generalisability of the HO-CTA model. The Effect Strength for Sensitivity (ESS) was used to assess predictor performance in classification of IFG. ESS is an index of effect strength normed against chance, where 0 represents classification accuracy expected by chance and 100 represents errorless classification [30]. Analysis was carried out using IBM SPSS (Version 22 for Windows, IL, USA), UniODA (American Psychological Association, USA) and CTA (Optimal Data Analysis LLC, USA) statistical packages. Level of significance was set at 0.05.

## Results

### Baseline characteristics of study population

Table 1 on characteristics of the study population shows that 2447 participants were selected consisting of 1151 males and 1296 females. The age (*p* = 0.700) and fasting blood glucose levels (*p* = 0.377) of participants were relatively the same across gender.

**Table 1:** Baseline characteristics of study population

	All population *N* = 2447	Female *N* = 1296	Male *N* = 1151	*p*-values
Age (years)	43.4 ± 14.8	43.3 ± 14.6	43.4 ± 15.1	0.700
Weight (Kg)	65.3 ± 18.1	64.6 ± 21.8	66.0 ± 12.5	<0.0001
Height (m)	1.6 ± 0.08	1.6 ± 0.07	1.7 ± 0.08	<0.0001
WC (cm)	84.0 ± 10.9	85.1 ± 11.9	82.9 ± 9.7	<0.0001
BMI (Kg/m^2^)	24.8 ± 6.2	25.6 ± 7.6	24.1 ± 4.0	<0.0001
WHtR	0.52 ± 0.07	0.54 ± 0.07	0.50 ± 0.06	<0.0001
SBP (mmHg)	122.2 ± 20.4	121.1 ± 21.3	123.5 ± 19.3	<0.0001
DBP (mmHg)	78.1 ± 14.2	77.4 ± 15.6	78.8 ± 12.3	<0.0001
TC (mg/dL)	167.5 ± 52.9	173.5 ± 56.1	160.7 ± 48.1	<0.0001
HDL (mg/dL)	50.9 ± 16.8	52.7 ± 17.5	48.7 ± 15.8	<0.0001
TG (mg/dL)	120.0 ± 49.4	117.1 ± 45.5	123.2 ± 53.3	0.042
FBG (mg/dL)	79.9 ± 30.0	79.7 ± 28.9	80.0 ± 31.3	0.377

*WC* waist circumference, *BMI* body mass index, *WHtR* waist to height ratio, *SBP* systolic blood pressure, *DBP* diastolic blood pressure, *TC* total cholesterol, *HDL* high density lipoprotein cholesterol, *TG* triglycerides, *FBG* fasting blood glucose

### Prevalence of co-morbid states of IFG and diabetes

The overall prevalence of IFG (5.8%; CI: 4.9 – 6.7%) was higher than that of diabetes (3.1%; CI: 2.4 – 3.8%). The highest co-morbid risk factor with IFG was dyslipidaemia (5.0%; CI: 4.1 – 5.8%), followed by overweight/obese (3.1%; CI: 2.5 – 3.8%), then HBP (1.8%; CI: 1.3 – 2.4%). In diabetes, the prevalence of co-morbid conditions followed the same trend as in IFG, where co-morbidity with dyslipidaemia (2.7%; CI: 2.0 – 3.3%) was the highest and HBP, the lowest prevalent (1.3%; CI: 0.9 – 1.8%) (Table 2).

**Table 2:** Prevalence of IFG and diabetes co-morbidity with CVD risk factors

CVD risk factors	Proportion (*N* = 2447)	Prevalence (%)	95% confidence interval		SE
			Lower	upper	
HBP	566	23.1	21.5	24.8	0.8
Hypercholesterolaemia	634	25.9	24.2	27.7	0.9
Hypertriglyceridaemia	572	23.4	21.6	25.1	0.9
Low HDL-C	1073	43.8	41.8	45.7	1.0
Overweight/obese	979	40.0	38.0	42.1	1.0
IFG	141	5.8	4.9	6.7	0.5
IFG + dyslipidaemia	122	5.0	4.2	5.9	0.4
IFG + HBP	64	2.6	2.0	3.3	0.3
IFG + overweight/obese	76	3.1	2.5	3.8	0.4
Diabetes	76	3.1	2.4	3.8	0.4
Diabetes + dyslipidaemia	65	2.7	2.0	3.4	0.3
Diabetes + HBP	47	1.9	1.4	2.5	0.3
Diabetes + overweight/obese	48	2.0	1.4	2.6	0.3

*IFG* impaired fasting glucose, + with, *HDL* high density lipoprotein cholesterol, *HBP* high blood pressure, *SE* standard error

### Co-morbidity of IFG and diabetes with other CVD risk factor across age groups

Across the different age groups, the results show that co-morbidity of IFG and dyslipidaemia was dominant more than the other risk conditions (Table 3). The lowest age group, (18 – 29 years) also had a sizeable prevalence of IFG and its co-morbid risk factors. The age group, 40 – 49 years had the highest proportion of individuals with risk conditions both in IFG and diabetes.

**Table 3:** Prevalence of IFG and diabetes co-morbidity with other CVD risk factors across age groups

Age Group(years)	IFG co-morbidity with CVD risks				Diabetes co-morbidity with CVD risks			
	IFG *n* (%)	IFG with dyslipidaemia *n* (%)	IFG with HBP *n* (%)	IFG with overweight/obese *n* (%)	Diabetes *n* (%)	Diabetes with dyslipidaemia *n* (%)	Diabetes with HBP *n* (%)	Diabetes with overweight/obese *n* (%)
18-29	30 (5.3)	24 (4.2)	9 (1.6)	11 (1.9)	12 (2.1)	11 (1.9)	5 (0.9)	8 (1.4)
30-39	21 (4.2)	19 (3.8)	4 (0.8)	13 (2.6)	9 (1.8)	8 (1.6)	3 (0.6)	5 (1.0)
40-49	35 (6.9)	29 (5.8)	13 (2.6)	19 (3.8)	19 (3.8)	16 (3.2)	10 (2.0)	12 (2.4)
50-59	25 (5.8)	23 (5.3)	11 (2.6)	17 (4.0)	16 (3.7)	13 (3.0)	8 (1.9)	10 (2.3)
60-69	28 (8.0)	25 (7.2)	7 (2.0)	15 (4.3)	11 (3.2)	9 (2.6)	6 (1.7)	10 (2.9)
70-89	2 (2.1)	2 (2.1)	1 (1.1)	1 (1.1)	9 (9.6)	8 (8.5)	1 (1.1)	3 (3.2)
Total	141 (5.8)	122 (5.0)	45 (1.8)	76 (3.1)	76 (3.1)	65 (2.7)	33 (1.3)	48 (2.0)

### Predicted age of IFG and diabetes co-morbidity with other CVD risk factors

There was no statistical significant difference between predicted age of participants with or without IFG, and IFG co-morbidity with dyslipidaemia and HBP (Table 4). On the other hand, only diabetes (p = 0.018) and its co-morbidity with HBP (p = 0.010) showed a statistical significant difference. In all, predicted age of IFG, diabetes and their co-morbid risk conditions were between 40 – 45 years old.

**Table 4:** Age (years) of IFG and diabetes co-morbidity with CVD risk factors

CVD risk factors	Status	Predicted age (years)	*N*	% satisfied	ESS	*p*-value
IFG	Yes	≥40	1376	6.54	8.06	0.253
	No	<40	1071	4.76		
IFG with Dyslipidaemia	Yes	≥41	1274	5.97	10.77	0.091
	No	<41	1173	3.92		
IFG with HBP	Yes	≥41	1274	2.43	17.14	0.096
	No	<41	1173	1.19		
IFG with Overweight/Obese	Yes	≥41	1274	4.00	15.52	0.033
	No	<41	1173	2.13		
Diabetes	Yes	≥40	1376	4.00	16.65	0.018
	No	<40	1071	1.96		
Diabetes with Dyslipidaemia	Yes	≥44	1169	3.51	15.72	0.052
	No	<44	1278	1.88		
Diabetes with HBP	Yes	≥40	1376	1.82	19.79	0.010
	No	<40	1071	0.75		
Diabetes with Overweight/Obese	Yes	≥42	1255	2.63	17.81	0.063
	No	<42	1192	1.26		

*N* number of observations in a given predicted class category, *% satisfied* percentage of observations in a given class category, *ESS* effect strength for sensitivity, *HBP* high blood pressure

### Classification tree of IFG co-morbid risk factors

**Fig. 1:**
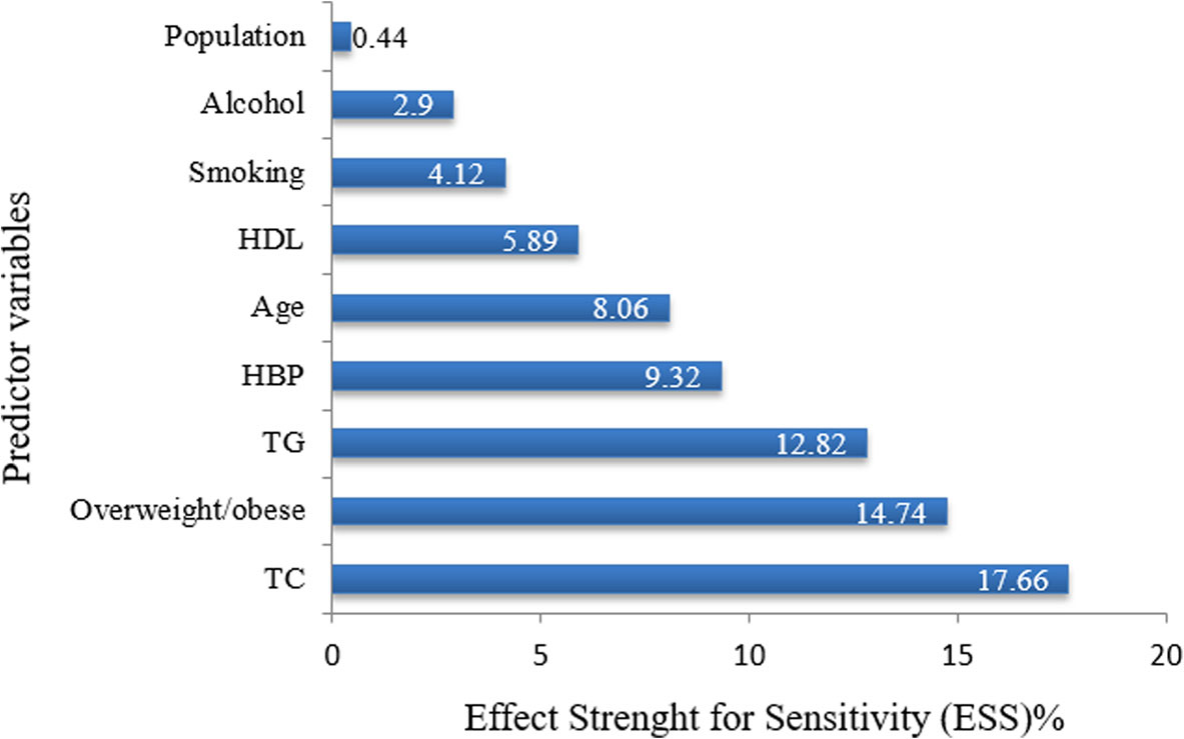
Order of independent predictive performance of the risk factors in classifying IFG. TC = elevated total cholesterol, TG = hypertrglyceridemia, HDL = High density lipoprotein cholesterol. Overweight/obese is based on body mass index, population = rural *vs* urban population

Figure 1 shows the order of independent predictive performance of the risk factors in classifying IFG. The highest and statistically significant independent predictors of IFG was elevated blood total cholesterol level (ESS = 17.66%, *p* < 0.0001), followed by overweight/obese (ESS = 14.74%, *p* = 0.001), hypertriglyceridaemia (ESS = 12.82%, *p* = 0.001) and high blood pressure (ESS = 9.32%, *p* = 0.013). However, age (ESS = 8.06%, *p* = 0.248), low blood HDL-C level (ESS = 5.89%, *p* = 0.189), cigarette smoking (ESS = 4.12%, *p* = 0.292), alcohol consumption (ESS = 2.90%, *p* = 0.609) and population (rural vs. urban) [ESS = 0.44%, *p* = 0.930] were not statistically significant.

**Fig. 2:**
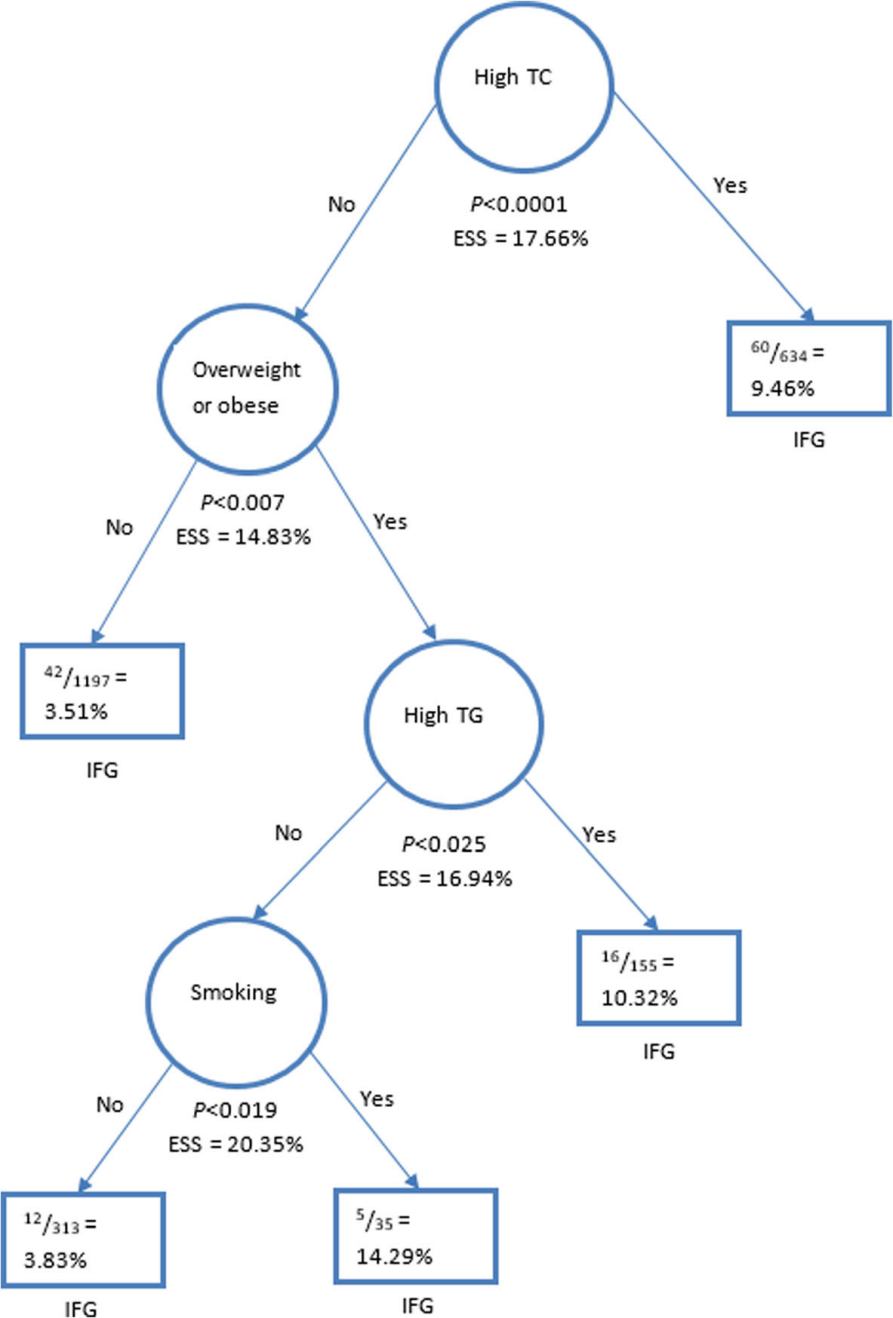
Hierarchically Optimal Classification Tree Analysis Model (via UniODA) of IFG subjects with risk factors. High TC = high level of total cholesterol; high TG = high level of triglyceride; overweight/obese is based on body mass index; ESS = effect strength for sensitivity

The classification tree model shown in Fig. 2 also indicates that high level of blood total cholesterol concentration is the most prominent/important co-morbid risk factor among IFG subjects. The result further shows that among 141 (5.8%) subjects with IFG, 60 (9.5%) have elevated total cholesterol. Among 81 (4.5%) IFG subjects without high level of total cholesterol, 39 (6.3%) are overweight/obese. Hypertriglyceridaemia is a risk factor in subjects with IFG and are overweight/obese even if they do not have elevated blood level of total cholesterol.

## Discussion

This study shows that there is higher prevalence of IFG than undiagnosed diabetes among the study Nigerian population. Further, co-morbidity of modifiable risk factors for chronic diabetes and CVD, such as dyslipidaemia and HBP, were more predominant in IFG than diabetes subjects. Several studies report that the transition to westernised ways of life, which may lead to unhealthy eating and physical inactivity, is possibly responsible for this increase in multi-morbidity [33–35]. It has been shown that the relative risk of coronary heart disease in individuals with 1, 2 or >3 CVD risk factors is up to 2, 4 and 7-folds, respectively [14]. More so, the fact that the hyperglycaemic condition of IFG worsens the already frail state of endothelial dysfunction and insulin resistance [4], prompts for action on community-wide implementation of diabetes and CVD risk factor identification and prevention.

Age distribution of IFG and co-morbidity of IFG with CVD risk factors showed upward trend from the lowest age group, plateauing at 40 – 49 years. This finding agrees with other studies on independent CVD risk factors, which showed an increase in prevalence with increase in age [36, 37]. No significant predicted age difference was observed in population with and without IFG and IFG co-morbidity with risk factors, except in overweight/obese. In other words, IFG or its co-morbid risk condition do not have age preference unlike in diabetes where the age difference was found to be statistically significant (p < 0.018) between age of occurrence (Table 4). However, the fact that co-existence of other risk factors with impaired glucose regulation increases the risk of chronic diabetes and CVD [38], underscores the need for frequent risk assessment for prediabetes in this population. Establishment of opportunistic screening strategies using already available communicable disease screening centres would be a cost-efficient approach.

Our findings also showed that predicted age of IFG, diabetes and their co-occurrence with other risk factors were within same range (40 – 45 years). Evidence shows that risk assessment models for CVD have diabetes as dichotomous (Yes or No) and considers subjects from 40 years of age. It is argued that the problem of such dichotomy is that hyperglycaemia in impaired fasting glucose or impaired glucose tolerance is missed via a NO answer, when assessment and treatment of CVD risk in prediabetes should be as in diabetes [39, 40]. The implication is that information pertaining to the primordial forms of glucose dysregulation are not brought to fore in Nigeria to aid early identification of the ‘at risk’ population, since risk of an individual is usually not assessed until overt diabetes that surfaces with huge symptoms. This emphasises that intervention based on outcome of utilisation of available risk assessment tools would not identify a substantial proportion of the population at increased risk of CVD, given the proportion of IFG individuals with co-morbid risk factors.

Furthermore, our findings delineated high level of blood total cholesterol as the most important risk factor predominant in population with IFG. Some studies in Nigeria identified high prevalence of risk factors [41–43], and insight into the order of importance and occurrence would assist in screening and treatment of the at-risk individuals. However, based on chain of events as shown in Figs. 1 and 2, it may seem important to integrate screening for hypertriglyceridaemia in risk assessment models for prediabetes conditions in the Nigerian population, as it strongly contributes to the co-morbidity in IFG-normocholesterolaemic-overweight/obese-nonhypertensive individuals. This implies that in the absence of HBP, there is increased chance of dyslipidaemia (high total cholesterol and/or triglyceride levels) and overweight/obese among the sample population with IFG. Furthermore, hypertriglyceridaemia was found to have higher independent predictive ability in classifying IFG than age. It was opined to be an essential risk factor contributing to discordance of results from the use of the ATPIII/Framingham risk score and metabolic syndrome in screening individuals at 10-year risk of CVD [44].

The increasing identification of IFG and undiagnosed diabetes is an issue for concern, requiring concerted and pragmatic approach. It is important to emphasise the need for screening of IFG at least, based on the evidence of high co-morbid CVD risk factors associated with it. The feasibility of screening by measuring FBG levels leads to increased chances of early identification when targeted at patients with multiple risk factors [45]. The epidemiological evidence that the majority of cases of diabetes/CVD are undiagnosed in this part of the world is an indication that assessment and management of CVD risk based on screening for diabetes in subjects with high blood pressure [46] may miss out early identification of other risk factors that co-exist in individuals without evidence of hypertension. This is on the premise that the etiological mechanisms of clustering of the CVD risk factors are not well understood. However, one of the factors undermining risk screening of dyslipidaemia in Nigeria is the high cost [20, 47], and although obesity screening is affordable, there is no evidence regarding its assessment against mortality outcome other than metabolic outcome, and in most cases the proportion of metabolically healthy obese phenotype is not accounted for in studies reporting high prevalence of obesity. Therefore, future studies exploring association of these phenotypes with prediabetes and other risk factors are required to elucidate the order of magnitude of IFG/IGT co/multi-morbidity with CVD risk factors in sub-Sahara Africa.

### Strength and Limitations of the study

One of the strength of the study is that it comprises of treatment naïve individuals for the assessed risk factors. Also, to the best of our knowledge, this is the only published study that has investigated the co-morbidity of IFG with other modifiable CVD risk factors and the order of importance of the IFG co-morbid risk factors in apparently healthy Nigerians. The use of a nonlinear multivariate classification method that is not influenced by assumptions of multicolinearity, multivariate normality, equality of group sizes, or number of variables is an added strength. The point of care analysis was carried out within manufacturers recommended operational temperature range, and this was confirmed by device logs used.

Diabetes mellitus is known to be heterogeneous, where differences in glucose regulation exist among individuals with comparable diagnostic glucose levels [48]. One of the limitations of this study is use of FBG measurement to classify diabetes. Although cost prohibitive it would be worth using HbA1c, which is now available as a point of care test as an additional screening tool.

## Conclusion

The higher prevalence of co-morbidity of CVD risk factors with IFG than in diabetes mellitus shows that targeting only the older adult individuals presenting at health institutions for risk factors identification and intervention would not abate the increasing incidence of diabetes and CVD in Africa. There is need for appropriate risk assessment models for prediabetes considering that IFG and its co-morbid risk factors are relative prevalent in young adults and probably more in middle-aged adults.

## Additional file

Additional file 1: Result of the Optimal Discriminant Analysis via UniODA. (TXT 24 kb)

## Abbreviations

ATPIII: Third adult treatment panel
BMI: Body mass index
CVD: Cardiovascular disease
DBP: Diastolic blood pressure
ESS: Effect strength for sensitivity
FBG: Fasting blood glucose
HbA1c: Glycated haemoglobin
HBP: High blood pressure
HDL-C: High density lipoprotein cholesterol
HO-CTA: Hierarchical optimal classification tree analysis
HREC: Human research ethics committee
IFG: Impaired fasting glucose
IGT: Impaired glucose tolerance
Kg/m^2^: Kilogram per meter squared
Mg/dL: Milligram per decilitre
mmHg: Millimetre hydrogen
mmol/L: Millimoles per litre
ODA: Optimal discriminant analysis
SBP: Systolic blood pressure
T2D: Type 2 diabetes
TC: Total cholesterol
TG: Triglyceride
